# Multicomponent DNA Nanomachines for Amplification-Free Viral RNA Detection

**DOI:** 10.3390/ijms26083652

**Published:** 2025-04-12

**Authors:** Valeria V. Solyanikova, Daria A. Gorbenko, Valeriya V. Zryacheva, Anna A. Shtro, Maria S. Rubel

**Affiliations:** 1 DNA-Nanosensoric Diagnostic Lab., ITMO University, 9 Lomonosova St., 191002 St. Petersburg, Russia; v_solyanikova@mail.ru (V.V.S.); daryarogova7@gmail.com (D.A.G.); msrubel@itmo.ru (M.S.R.); 2 Smorodintsev Research Institute of Influenza, 15/17 Prof. Popova St., 197022 St. Petersburg, Russia; Valerya-zr4@yandex.ru; 3 Amyloid Biology Lab., St. Petersburg State University, 7-9 Universitetskaya enb., 199034 St. Petersburg, Russia

**Keywords:** point-of-care diagnostics, amplification-free detection, DNA nanosensors, multicomponent DNA nanomachines, viral RNA, DNAzymes

## Abstract

The rapid and accurate detection of viral infections is of paramount importance, given their widespread impact across diverse demographics. Common viruses such as influenza, parainfluenza, rhinovirus, and adenovirus contribute significantly to respiratory illnesses. The pathogenic nature of certain viruses, characterized by rapid mutations and high transmissibility, underscores the urgent need for dynamic detection methodologies. Quantitative reverse transcription PCR (RT-qPCR) remains the gold-standard diagnostic tool. Its reliance on costly equipment, reagents, and skilled personnel has driven explorations of alternative approaches, such as catalytic DNA nanomachines. Diagnostic platforms using catalytic DNA nanomachines offer amplification-free nucleic acid detection without the need for protein enzymes and demonstrate feasibility and cost-effectiveness for both laboratory and point-of-care diagnostics. This study focuses on the development of multicomponent DNA nanomachines with catalytic proficiency towards a fluorescent substrate, enabling the generation of a fluorescent signal upon the presence of target nucleic acids. Specifically tailored variants are designed for detecting human parainfluenza virus type 3 (HPIV) and respiratory syncytial virus (RSV). The engineered DNA nanomachine features six RNA-binding arms for recognition and unwinding of RNA secondary structures, along with a catalytic core for nucleic acid cleavage, indicating potential utility in real clinical practice with minimal requirements. This research showcases the potential of DNA nanomachines as a reliable and sensitive diagnostic tool for RNA virus identification, offering promising prospects for clinical applications in the realm of infectious disease management.

## 1. Introduction

The rapid and accurate identification of viral infections is vital for effectively controlling and containing epidemics. The foremost step in addressing viral infections is to obtain a precise and prompt diagnosis [1]. The identification of the particular pathogen that is responsible for diseases such as pneumonia is pivotal in determining the suitable therapy and epidemiological spread of the pathogen.

The majority of acute respiratory disease episodes are attributable to viral infections, including parainfluenza virus (HPIV), respiratory syncytial virus (RSV), rhinovirus, influenza virus, and adenovirus [2]. RSV, HPIV, rhinovirus, and coronavirus are common in both minor and adult patients with community-acquired pneumonia. The HPIV triggers concerns and poses a significant threat to infants and children, as it can cause croup and the rapid development of serious complications in the upper and lower respiratory tract [3]. In immunocompromised individuals, these infections can cause alternate severe effects and should therefore be accurately discriminated from common colds and other similarly manifested diseases at as early a stage as possible. Moreover, detecting a specific pathogen of the influenza virus, HPIV, or RSV in hospitalized individuals can aid in averting potential outbreaks [3,4].

Currently, the main diagnostic method remains quantitative reverse transcription polymerase chain reaction (RT-qPCR). This method can detect even low levels of virus but requires specific equipment, reagents, as well as qualified personnel [5,6]. Researchers have suggested new approaches [7], mostly connected to microfluidic and isothermal amplification [8]. DNA nanosensors are an alternative, cutting-edge diagnostic option that can be adapted to viral nucleic acid detection. DNA nanosensors hold promise for point-of-care diagnostics in resource-limited settings, where access to specialized equipment and trained personnel is restricted. Their amplification-free operation and rapid turnaround time (15–60 min) make them suitable for decentralized testing during viral outbreaks or in remote regions.

This research presents two different diagnostic approaches based on DNA nanosensors and successfully applies them to viral ribonucleic acid identification. We use (1) binary molecular beacon-based DNA nanosensors (MB-DNSs) coupled with initial nucleic acid accumulation via RT-PCR and (2) multicomponent DNA nanosensors, called DNA nanomachines (DNMs), with a deoxyribozyme core that are used without amplification.

The MB-DNS does not require a long incubation time or specific temperature conditions [9]. The biggest drawback of this sensor type is its sensitivity limit within 1 nM [9,10,11]. Earlier studies show that this level is enough for the amplification-free detection of bacterial rRNA [10], but to adapt them for a low abundance of viral samples, we have added the amplification step. The research employs a molecular beacon based on the concept of a closed loop architecture, which undergoes unfolding when exposed to complementary segments of a binary probe (see MB, Table S1).

To overcome the problem of detection sensitivity and make the test amplification-free, we elevated the previously developed concept of a catalytic DNM. A DNM is an all-oligonucleotide construction that possesses catalytic activity due to a formation of a deoxyribozyme core [9,12]. Deoxyribozyme 10–23 is a DNA fragment that forms a specific 3D structure, which cleaves the reporter substrate, generating a fluorescent signal. The design of a multicomponent DNM was equipped with additional fragment-binding sequences (arms) and an additional double-stranded DNA tile. According to earlier research, such a complex multicomponent design is capable of more successful recognition and unwinding of secondary structures and even double-stranded (ds) DNA [13,14,15,16]. The design was also improved by adding a hook strand, which binds to the DNA tile and is partially complementary to the fluorescent substrate [17]. The hook strand, being a part of the DNM, attracts F-sub from the solution and increases its local concentration around the deoxyribozyme core, which subsequently increases the signal turnover [17]. In this work, we compare the performance of our improvements with the previously described concepts [18].

The purpose of this research was to develop diagnostic concepts for viral single-stranded RNA detection based on binary MB-DNSs and multicomponent DNMs and compare the feasibility of their application for viral RNA detection.

## 2. Results

### 2.1. Selection of RNA Fragments

We identified unique conserved regions of viral genomic RNA through NCBI database analysis (GenBank: MH678683.1; OQ171930.1). Target sequences were selected based on UNAfold secondary structure predictions [19], prioritizing regions with minimal self-complementarity and optimal accessibility characteristics. This approach maximized the hybridization efficiency and ensured specificity in our DNA nanosensor design.

### 2.2. Design of DNA Nanosensors

**Figure 1:**
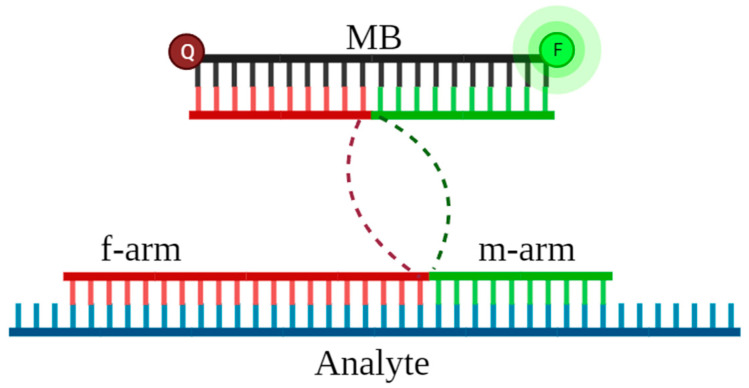
MB-DNS structure. The dotted lines indicate the hexaethyleneglycol (HEG) linkers that connect the analyte-binding and molecular beacon (MB)-binding parts of the sensor’s arms; F—FAM; Q—Black Hole Quencher.

MB-DNSs consist of two arms with RNA-binding parts, which are complementary to the selected RNA sequence, and two reporter-binding parts that are complementary to the molecular beacon (MB, see Table S1) (Figure 1). One of the arms (the f-arm in Figure 1) intentionally remains long to promote the binding to a hairpin of the analyte.

**Figure 2:**
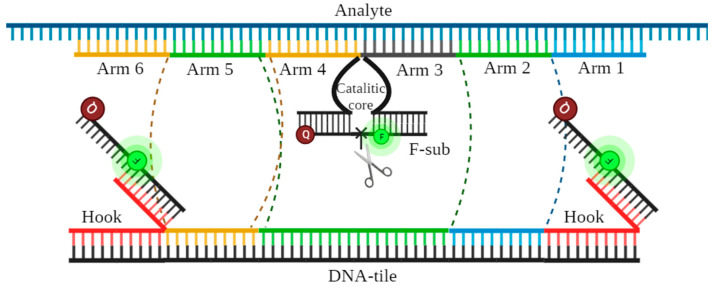
Structure of the 6-arm DNMs. The dotted lines indicate the linkers that connect the analyte-binding parts of the arms and DNA tile; F—FAM; Q—Black Hole Quencher. The scissors schematically indicate the location of the cleavage site of the fluorescent substrate (F-sub).

Each DNA nanomachine (DNM) consists of RNA-binding arms that provide highly specific binding of the nanomachine and RNA in accordance with the principle of complementarity. The DNM also contains a dsDNA tile that contributes to the stabilization of the DNM and its improved assembly in the presence of viral RNA. The central arms, upon the complete assembly of the 6DNM, form the deoxyribozyme catalytic core, which has specific catalytic activity and can selectively “cut” the fluorescent substrate (F-sub, see Table S2) sequence that contains the fluorophore and quencher. Thus, by cutting the fluorescent substrate, the quencher and the fluorescent agent are separated from each other, and the fluorescent signal is released (Figure 2).

To compare the performance of the newly developed six-arm DNM concept (6DNM), a four-arm DNM (4DNM) that was previously described in [18] was also designed for the same sequence region and characterized (Figure S3).

### 2.3. Detection by MB-DNSs

#### 2.3.1. Estimation of LOD on a Synthetic Analyte

**Figure 3:**
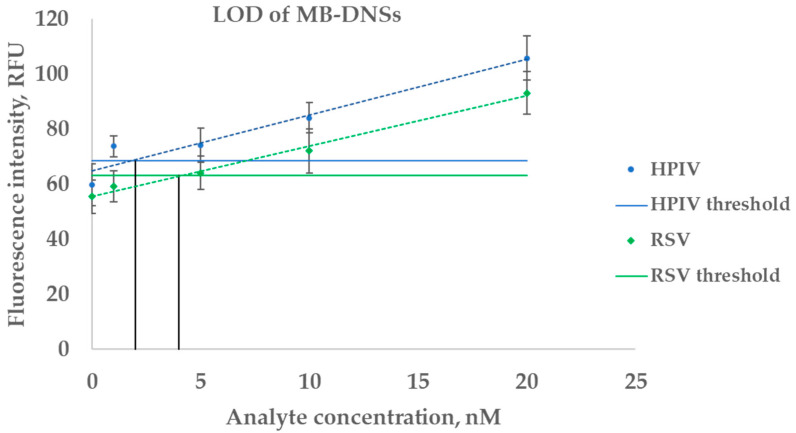
Limits of detection of MB-DNSs. The blue color corresponds to the HPIV analyte detection results, and the green color corresponds to the RSV analyte detection results. The dotted lines represent the linear trendline. The black lines indicate the detection limits for each MB-DNS. Data represent mean ± SD of triplicate experimental measurements.

Detection using MB-DNSs was performed on synthetic oligonucleotides with a 15 min incubation at room temperature. The developed binary RSV and HPIV MB-DNSs demonstrated limits of detection (LODs) of 2 nM and 4 nM, respectively (Figure 3). While MB-DNSs effectively detect analyte concentrations ranging from 10 to 100 nM and above, this sensitivity is insufficient for the direct detection of viral genomic RNA without amplification. Consequently, our subsequent experiments utilized MB-DNSs in conjunction with RT-PCR amplicons to achieve clinically relevant detection thresholds.

#### 2.3.2. RT-PCR Amplicon Detection Results

**Figure 4:**
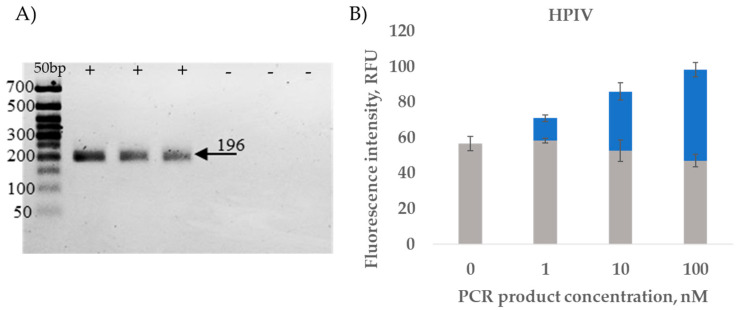
PCR product detection by HPIV MB-DNS. (**A**) Visualization of HPIV fragment (196 bp): 50+ bp—50+ bp DNA Ladder (Evrogen); “-”—negative control; “+”—experimental tubes. (**B**) PCR product detection with HPIV MB-DNS. The gray color shows the fluorescence values of the negative PCR control, while the blue color shows the detection signal of the PCR product. Data represent mean ± SD of triplicate experimental measurements.

**Figure 5:**
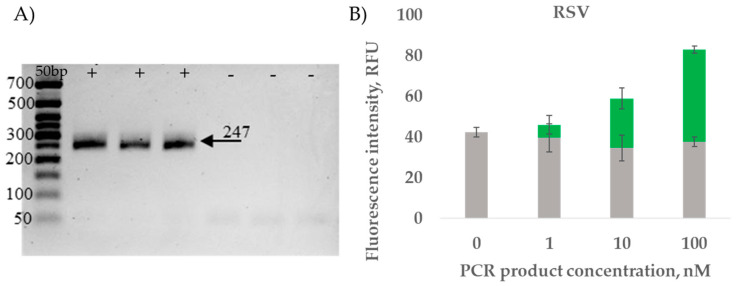
PCR product detection by RSV MB-DNS. (**A**) Visualization of RSV fragment (247 bp): 50 bp—50+ bp DNA Ladder (Evrogen); “-”—negative control; “+”—experimental tubes. (**B**) PCR product detection with RSV MB-DNS. The gray color shows the fluorescence values of the negative PCR control, while the green color shows the detection signal of the PCR product. Data represent mean ± SD of triplicate experimental measurements.

To assess the sensitivity of the RT-PCR amplification and the reaction detection, serial dilution of HPIV and RSV RT-PCR products was performed. The amplification products were analyzed in 2% agarose gel (Figure 4A and Figure 5A). The amplicons of different concentrations (1 nM, 10 nM, 100 nM) were detected, which confirmed the possibility of specific identification of amplified viral RNA (Figure 4B and Figure 5B).

Our results demonstrate that binary DNA nanosensors effectively detect RT-PCR products after denaturation. The produced fluorescence signal is easily distinguished and is two times higher in the case of a PCR product concentration of 100 nM.

#### 2.3.3. Clinical Sample Detection Results

**Figure 6:**
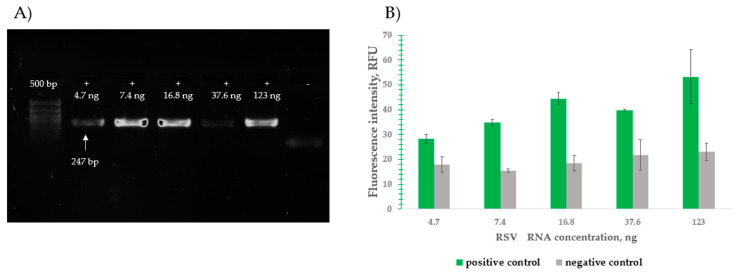
RT-PCR product detection by RSVA MB-DNS. (**A**) Visualization of RSVA fragment (247 bp): 50+bp—50+ bp DNA Ladder (Evrogen); “-”—negative control; “+”—experimental tubes. (**B**) PCR product detection with RSV MB-DNS. The gray color shows the fluorescence values of the negative PCR control, while the green color shows the detection signal of the PCR product. Data represent mean ± SD of triplicate experimental measurements.

Five clinical RSV samples were obtained from the Institute of Influenza, Russia. Total RNA was extracted using a phenol–chloroform protocol, yielding concentrations of 4.7, 7.4, 16.8, 37.6, and 123 ng (Figure 6A). Following extraction, all specimens underwent RT-PCR amplification and subsequent MB-DNS detection. The analytical method successfully identified RSV in all five samples, with four samples’ signal-to-background ratios consistently exceeding 2.5, demonstrating robust detection capabilities across varying viral loads (Figure 6B).

The use of binary MB-based DNA nanosensors as a substitute for visualizing standard PCR outcomes displays promising potential. These sensors possess the ability to identify specific products within a short incubation time without special temperature conditions. The detection experiments demonstrated that comparable concentrations of synthetic DNA and single-stranded (ss) PCR products result in equivalent fluorescence.

### 2.4. Detection by DNMs

#### 2.4.1. DNM Assembly Visualization

**Figure 7:**
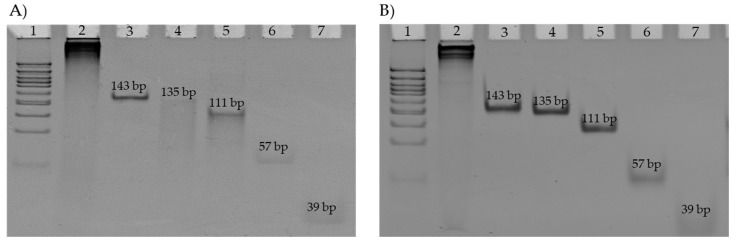
PAGE (8%) of 6DNM assembly: (**A**) HPIV 6DNM assembly; (**B**) RSV 6DNM assembly. 1—50+bp DNA ladder; 2—6DNM; 3—DNA tile (143 bp); 4—a2t2a5 strand (135 bp); 5—a2t3a6 strand (111 bp); 6—a1t1 strand (57 bp); 7—hook strand (39 bp).

The 6DNM tile-part was assembled before the detection experiments and visualized in 8% native polyacrylamide gel (Figure 7). This pattern suggested incomplete hybridization stability among the machine components, with gradual dissociation occurring during electrophoresis, resulting in the observed additional bands. Notably, the absence of bands corresponding to individual arm sizes confirmed successful machine assembly.

Prior to the detection experiments, the 4DNM tile component was separately assembled and verified using 8% native polyacrylamide gel electrophoresis (Figure S1). Both of the 4DNMs exhibited incomplete assembly, just as the 6DNMs, and the HPIV demonstrated less stable assembly.

#### 2.4.2. Estimation of LOD for Synthetic DNA

The limit of detection was determined for the developed DNM with synthetically synthesized DNA oligonucleotides corresponding to the sequence of the target RNA fragment after 1 h and 3 h of incubation. Before the estimation of LODs, the best arms ratio was found for both 6DNMs via titration assays (see Supplementary Materials, Figures S2 and S3, Table S5). All of the DNM detection experiments were conducted based on these ratios.

**Figure 8:**
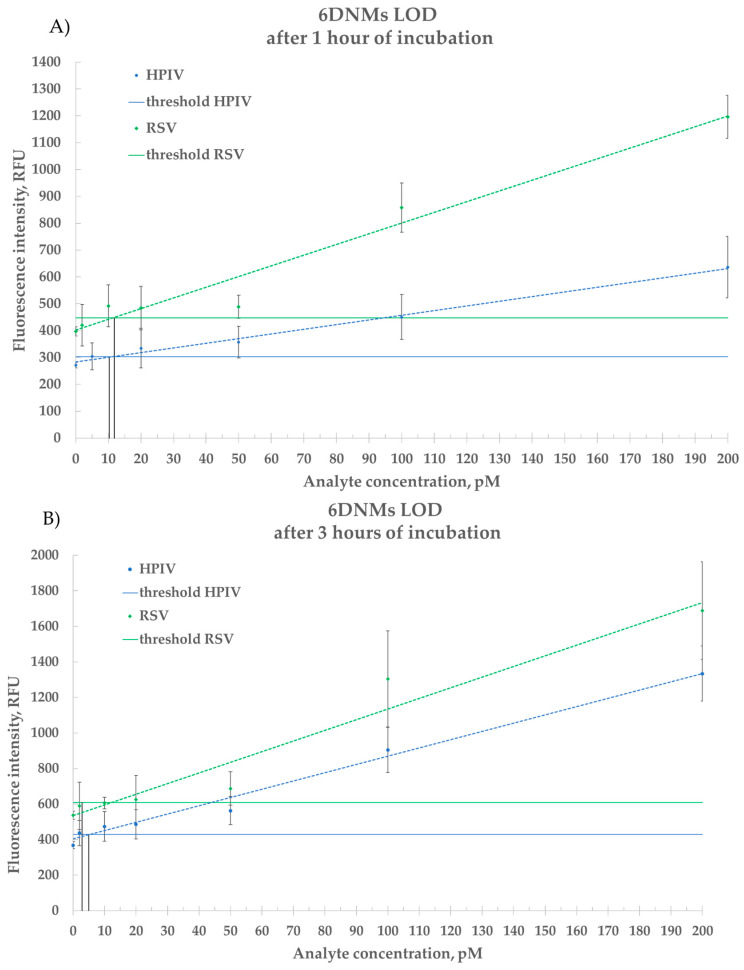
Estimated limit of detection for the synthetic analyte by 6DNMs. (**A**) HPIV and RSV 6DNMs after 1 h of incubation; (**B**) HPIV and RSV 6DNMs after 3 h of incubation. The blue color corresponds to the HPIV 6DNM result; the green color corresponds to the RSV 6DNM result. Dotted lines are the linear trendlines. The black lines indicate the detection limits for each DNM. Data represent mean ± SD of triplicate experimental measurements.

According to the results, the 6DNM’s HPIV LOD is 10 pM after 1 h and 5 pM after 3 h. The 6DNM’s RSV LOD is 12 pM after 1 h and 3 pM after 3 h (Figure 8).

Based on the obtained limits of detection results (Figure 8), it is suggested that the developed 6DNMs could perform amplification-free detection of total viral ssRNA isolated from viral particles.

Under identical conditions, the 4DNMs demonstrated comparatively lower sensitivities: their HPIV detection limits were 109 pM after 1 h and 43 pM after 3 h of incubation, while their RSVA detection limits were approximately 51 pM and 17 pM, respectively (Figure S4). These results indicate that 4DNMs are approximately 10-fold less sensitive than their 6DNM counterparts. Consequently, all subsequent experiments with clinical samples were conducted exclusively using the 6DNMs due to their superior detection capabilities

#### 2.4.3. Detection of Total Genomic RNA

Different concentrations of RNA were tested (100 fM, 200 fM, 500 fM, 1000 fM) during the RNA detection experiments.

**Figure 9:**
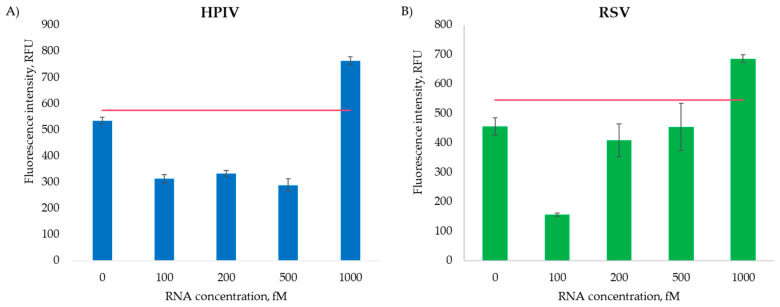
Viral RNA detection results. (**A**) HPIV RNA detection by the HPIV 6DNM; (**B**) RSV RNA detection by the RSV 6DNM. The red lines indicate the detection thresholds. Data represent mean ± SD of triplicate experimental measurements.

The results obtained for HPIV and RSV RNA detection (Figure 9) show that the 6DNM is able to specifically recognize viral RNA even in limited concentrations—at 1 pM. The detection capabilities of the RNA molecules are higher than those of the synthetic DNA. This is due to the greater hybridization affinity and stability of DNA-RNA hybrids in comparison to DNA-DNA hybrids [15].

The 6DNMs effectively detect RNA at a 1 pM concentration, although high background signals were observed, potentially due to the spontaneous degradation of a suboptimal F-sub batch during incubation, which may mask the true fluorescence signal. The fluorescence decrease at low RNA concentrations likely results from non-specific hybridization between the DNA nanomachine arms and the occasional formation of unintended deoxyribozyme cores (Figure 9). The observed reduction in RFU at the lowest RNA concentration may stem from non-specific interactions between sample components (e.g., residual extraction reagents or host nucleic acids) and the nanosensor. Such interactions could inhibit fluorescence signal generation or destabilize the DNA nanomachine assembly. Future studies might incorporate purification protocols (e.g., silica-membrane RNA cleanup) to mitigate interference.

Viral particle detection was hindered by the method’s limited dynamic range. Furthermore, the relatively low incubation temperature was insufficient to completely denature the RNA’s secondary structure. It is worth noting that extended incubation periods may lead to RNA degradation, consequently diminishing the signal intensity.

#### 2.4.4. Detection of RNA Extracted from Clinical Samples

**Figure 10:**
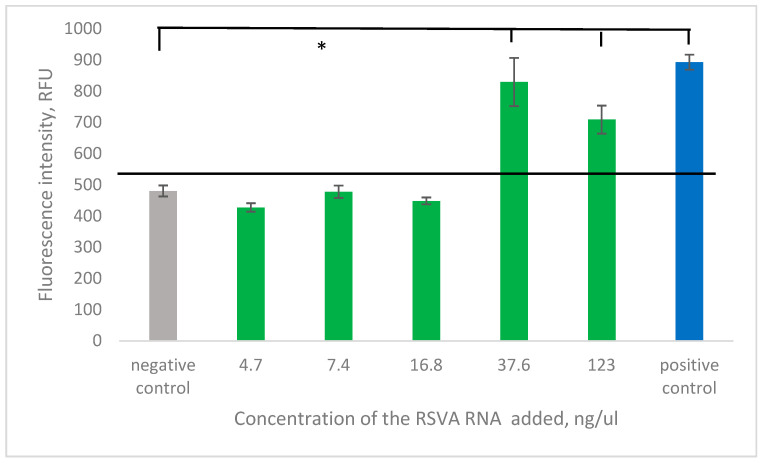
Detection of total DNA with 6DNMs, extracted from clinical samples. The gray bar is the negative control with no sample added. The green bars are the experimental samples. The concentration of a sample is denoted under the bar. The blue bar is the positive control with 1 nM of the synthetic analyte added. The black line is the threshold. * *p* < 0.05. Data represent mean ± SD of triplicate experimental measurements.

The same five RSVA samples from Section 2.3.3 were subjected to 6DNM detection. RNA was extracted using a phenol–chloroform protocol, and 30 μL of each sample was added to the reaction of detection. The 6DNM successfully detected and produced statistically significant results in only two out of the five samples (Figure 10).

This confirms that 6DNMs can detect clinical samples; however, the viral particle count and, consequently, the extracted RNA concentration must remain higher than the threshold that is suitable for RT-PCR combined with MB-DNS.

## 3. Discussion

This study investigates two methods for detecting target nucleic acid sequences in a solution and compares their feasibility for detecting viral RNA. Both of the proposed methods can complement existing approaches in resource- or time-limited settings, such as outbreak surveillance or point-of-care detection.

The use of MB-DNSs is a well-studied diagnostic technique, but their LODs may not be sufficient for detecting long and folded viral RNA without pre-amplification. MB-DNSs can be an alternative to visualizing amplification results, as they are able to specifically recognize products requiring no special temperature conditions and have a short incubation time. We suggest using MB-DNSs in a coupled format to assist existing conventional amplification techniques to ensure speed and convenience, as well as to increase selectivity to the amplicon and its nucleotide variations [20].

The sensitive multicomponent 6DNMs developed and tested in this work were found to be suitable for the diagnosis of viral nucleic acids without amplification. Previously, 4DNMs have demonstrated utility in bacterial rRNA detection and pre-amplified structured DNA recognition [13,16], but this study presents the first application of 6DNMs for amplification-free viral RNA detection. The reduced sensitivity of 4DNMs for HPIV compared to RSVA likely stems from differences in target RNA secondary structures and from the incomplete assembly of the HPIV 4DNM. HPIV genomic regions exhibit higher GC contents and more stable hairpins, which impede 4DNM hybridization. In contrast, the 6DNM’s additional binding arms and DNA tile enhance structural unwinding, mitigating this limitation. Future designs could further optimize the arm sequences for challenging targets.

While 6DNMs have shown promising, they are not as sensitive as amplification techniques. The observed variability in clinical sample detection highlights the challenge of low viral loads in real-world settings. False negatives could arise if RNA concentrations fall below the LOD, necessitating pre-concentration steps or optimized extraction protocols (Section 4.1.2). Future iterations may integrate RNA enrichment methods, as demonstrated in similar diagnostic platforms [17]. The precise parameters and protocol of the test can be outlined after its additional characterization.

The main advantage of DNMs are that they do not require a thermal cycling device or any initial amplification. Polymerases that are used in amplification tend to make mistakes in triphosphate integrations and can be subjective to certain sequences, such as having difficulties with the amplification of CG-enriched fragments. Both of these issues have been solved by amplification-free detection, with 6DNMs playing a role as merely simple hybridization probes. In addition, for complex targets (high GC contents, stable secondary structures), binary sensors are unlikely to demonstrate sufficient thermodynamic energy for effective unfolding and binding of the analyte. Diagnostics based on multicomponent DNMs can be considered as an alternative to classical amplification methods.

This study’s small sample size reflects practical constraints in accessing clinical materials during the research period. While preliminary, these results validate the feasibility of 6DNMs for viral RNA detection. Larger-scale validation is planned pending collaboration with clinical partners to secure additional samples.

The features and characteristics of the proposed methods are presented in Table 1. MB-DNSs were found to provide faster results (15 min) compared to deoxyribozyme sensors and 6DNMs (30 min to several hours) and regular RT-PCR (90–120 min). In addition, 6DNMs require pre-processing in the form of an assembly step. At the same time, multicomponent 6DNMs can achieve sensitivity even in the fM ranges, while MB-DNSs and previously published 4DNMs exhibit sensitivity in the tens of pMs [9,18]. The developed 6DNMs are capable of detecting viral ssRNA without amplification and the synthetic ssDNA analyte at concentrations lower than 10 pM. As a result, even at an RNA concentration of 100 fM, the 6DNM for HPIV demonstrated a distinguishable fluorescence signal. The specificity of MB-DNSs and 6DNMs has been shown earlier in a number of studies [21,22,23]. Both methods require RNA isolation. An additional cDNA-synthetizing kit and a thermal cycler are required for the MB-DNS assay and RT-qPCR, but not for DNMs. According to our estimation, the price of the MB-DNS and 6DNM do not differ much. But taking into account the preliminary treatment and accumulation of the fragment, the full RT-PCR-MB-DNS assay may become up to five times more expensive. Both assays presented here can be used in practical circumstances to effectively identify viral ssRNA in a solution. RT-qPCR remains the gold standard for sensitivity [24], but 6DNMs offer distinct advantages in scenarios with minimal infrastructure. For example, in rural healthcare facilities or during field surveillance, 6DNMs could provide preliminary screening, reducing the reliance on centralized laboratories, and when lacking thermal cyclers, 6DNMs could serve as rapid screening tools. Their amplification-free nature and minimal equipment needs [9,20] make them suitable for scenarios where speed and cost outweigh the need for ultra-high sensitivity. Further integration with portable fluorescence readers could enable real-time, on-site diagnosis. MB-DNS, in its turn, may complement conventional PCR in equipment-restricted laboratories, as it demonstrates an ability to selectively determine the presence of the analyte. Both techniques can be applied, depending on the capacity of the laboratory, the purpose of the test, and the initial assumed concentration of nucleic acid in the sample.

Both the DNMs and MB-DNSs require further improvements and modifications to create more feasible instrument-free readouts of the MB-DNS and more sensitive DNMs.

## 4. Materials and Methods

### 4.1. Materials

#### 4.1.1. Detection Assay

For detection implementation, the following reaction buffer was used: Col buffer (200 mM MgCl2 (Helicon, Moscow, Russia), 15 mM NaCl (Helicon, Moscow, Russia), 150 mM KCl (Helicon, Moscow, Russia), and 50 mM HEPES, pH = 7.4 (Helicon, Moscow, Russia)). All the oligonucleotides were purchased in Evrogen and DNA synthesis, as an individual order. Tables S1–S3 depict the sequences of the oligonucleotides used in this study. The oligonucleotides were dissolved in DNAse/RNAse-free water (Invitrogen, Carlsbad, CA, USA) and stored at −20 °C.

#### 4.1.2. RNA Extraction

RNA was extracted from the viral particles by Quick-RNA Viral Kit (Zymo research, Orange, CA, USA) or via a standard phenol–chloroform protocol. The extracted RNA was immediately used for reverse transcription and detection experiments or aliquoted and stored at −80 °C.

#### 4.1.3. RT-PCR

dNTPs (10 mM of each, Evrogen, Moscow, Russia), RNase-free MQ water (Invitrogen, Carlsbad, CA, USA), MMLV reverse transcriptase (Evrogen, Moscow, Russia), Taq polymerase (Evrogen, Moscow, Russia), and 10× Taq turbo buffer (Evrogen, Moscow, Russia) were used.

#### 4.1.4. Electrophoresis

Agarose LE 2 (Helicon, Moscow, Russia), ethidium bromide 0.5 µg/mL (Evrogen, Moscow, Russia), 100+ bp DNA ladder (Evrogen, Moscow, Russia), and 4x DNA loading buffer (Evrogen, Moscow, Russia) were used. MQ water was purified via Millipore RiOs-DI 3 Smart (Merck, Darmstadt, Germany) and used for buffers and solutions.

#### 4.1.5. Purification of PCR Products

The purification of the amplification products from primer residues, reaction components, and enzymes was carried out using the Cleanup Mini kit (Evrogen, Moscow, Russia).

### 4.2. Methods

#### 4.2.1. Objects of the Research

The objects of this study were respiratory viruses’ total genomic ssRNA, isolated from the following samples: Human parainfluenza virus type 3 (HPIV) and Human respiratory syncytial virus A (RSV). Viral particles samples from collection lines and clinical samples (from nasopharyngeal swabs) of strains 34478 and SPB23 were provided by the Laboratory of Chemotherapy for Viral Infections, Smorodintsev Research Institute of Influenza, Saint-Petersburg, Russia. All the materials were collected according to the rules of clinical sample management of the Russian Federation. The RNA extraction was performed using the Quick-RNA Viral Kit according to the manufacturer’s instructions or via the phenol–chloroform method. The concentration of viral RNA was calculated by measuring its absorption at 260 nm using a ND-50, Implen, München, Germany (the phenol–chloroform method suggests the extraction of total RNA). The RNA was stored at −80 °C, aliquoted, and used for RT-PCR and amplification-free detection of viral RNA with DNA nanomachines.

#### 4.2.2. Design of MB-DNSs and DNMs

For the implementation of DNA nanosensors and nanomachines, the NCBI database was explored, and the target virus genome regions were selected according to the following criteria: important coding region, conservativity, and allowance for amplification. The targets’ folding was analyzed using the UNAfold and NUPACK tools. Binary MB-DNSs and DNMs were developed according to their complementarity to the target. Descriptions of the design process can be found in earlier articles by our team [23] under Col buffer conditions.

#### 4.2.3. DNA Nanomachine Assembly

The assembly included annealing the DNM’s arms, hook, and tile in equimolar concentrations in a Eppendorf tube in the reaction Col buffer [2]. Annealing was performed by using a heating mantle with boiling water and letting it passively cool down overnight.

#### 4.2.4. Polyacrylamide Gel Electrophoresis

Polyacrylamide gel electrophoresis (PAGE) was used to visualize the assembled DNA nanomachines. The analysis of the DNA machine assembly was performed in 8% native polyacrylamide gel for 90 min at 80 V.

#### 4.2.5. RT-PCR

The primer’s design was carried out with the NCBI Primer BLAST web tool (https://www.ncbi.nlm.nih.gov/tools/primer-blast/ accessed on 4 March 2025) to the chosen viral genome regions. RT-PCR was performed in the Thermal cycler T100 (Bio-Rad, Hercules, CA, USA). The MMLV RT kit and Taq DNA polymerase, Evrogen, Moscow, Russia, were used for reverse transcription and PCR.

#### 4.2.6. Agarose Gel Electrophoresis

Gel electrophoresis (2%) was performed for the visualization of the RT-PCR products. Samples with a 4× loading dye and 50+bp DNA ladder were loaded to the gel wells and run at 100 V for 30 min.

#### 4.2.7. Purification of Double-Stranded DNA Fragments

The purification of the amplification products from primer residues, reaction components, and enzymes was carried out in order to obtain pure dsPCR products without affecting the detection signal. Amplicon purification was performed using the Cleanup Mini kit, Evrogen, Moscow, Russia, according to the manufacturer’s instructions.

#### 4.2.8. Fluorescent Detection Method: Detection by MB-DNSs

Detection by MB-DNSs was performed in 1.5 mL tubes. Arms were added in the reaction Col buffer with the concentration of the f-arm (HPIV_f1; RSV_f1) and m-arm (HPIV_m1; RSV_m1) being 1000 nM and 100 nM, respectively. Different synthetic analyte (HPIV_an; RSV_an) concentrations (0 nM, 1 nM, 10 nM, 100 nM, 500 nM, 1000 nM) were tested. The negative control tube that contained two MB-DNS arms (f-arm (HPIV_f1; RSV_f1) 1000 nM and m-arm (HPIV_m1; RSV_m1) 100 nM) and MB (20 nM) in a reaction Col buffer was considered a background signal value. Samples were incubated for 15 min at room temperature. In the case of PCR product detection, purification of the amplicons and several dilutions were performed. The amplicon concentration was estimated using the NanoPhotometer NP80, Implen, München, Germany. To obtain a single-stranded target, an additional denaturation step was introduced. Denaturation involves heating the PCR products up to 95 °C and incubating them for 5 min in order to obtain a single-stranded product. A negative PCR control was used as a negative control for the RT-PCR product detection experiment. The fluorescent output from each tube was measured using a Spark microplate reader, Tecan, Männedorf, Switzerland, at an excitation of 480 nm and emission of 525 nm. The threshold was calculated as the fluorescence from the negative control tube (background) plus three times the standard deviation of the background.

#### 4.2.9. Fluorescent Detection Method: Detection by Multicomponent DNMs

For the detection by multicomponent 6DNMs, the fluorescent substrate (200 nM), machine after assembly (20 nM), arm-3-core strand (HPIV_a3c; RSV_a3c) (20 nM), and synthetic analyte (HPIV_an6; RSV_an6) (1–1000 pM) or extracted viral RNA (1–1000 fM) were placed into test tubes with the Col buffer. A test tube with all the reaction components except the analyte (HPIV_an6; RSV_an6 or RNA) was a negative control. Test tubes were incubated at 55 °C for 1 and 3 h in a water bath. The fluorescent output from each tube was measured using a Spark microplate reader, Tecan, Switzerland, at an excitation of 480 nm and emission of 525 nm. The threshold was calculated as the fluorescence from the negative control tube (background) plus three times the standard deviation of the background. A *t*-test was used to compare the differences between the results.

#### 4.2.10. Limit of Detection

The limit of detection (LOD) is defined as the lowest quantity or concentration of a component that can be reliably distinguished from the background [24]. The LOD was calculated as the lowest analyte concentration producing a fluorescence signal exceeding the threshold (mean background + 3σ), with triplicate experimental measurements at each concentration. Raw data for measuring the detection limit can be found in the Supplementary Materials (Tables S6–S10).

The limit of detection for the binary MB-DNSs was calculated after 15 min of sample incubation at room temperature. The test tubes contained different analyte (HPIV_an; RSV_an) concentrations: 0 nM, 5 nM, 10 nM, 15 nM, and 20 nM. The negative control tube that contained two MB-DNS arms (f-arm (HPIV_f1; RSV_f1) 1000 nM and m-arm (HPIV_m1; RSV_m1) 100 nM) and MB (20 nM) in a reaction Col buffer was considered a background signal value.

For the experiment with 6DNMs, the LOD assay involved incubating the best machine’s component concentration, which was determined during the titration assay (Supplementary Materials, Figures S1 and S2, Table S4) in the reaction Col buffer with various concentrations of the synthetic analyte (HPIV_an6; RSV_an6)—2 pM, 5 pM, 10 pM, 20 pM, 50 pM, 100 pM, 200 pM, 500 pM, and 1000 pM—and fluorescent substrate (200 nM). The fluorescence produced from a sample with all components except an analyte in the reaction buffer was used as a background. All tubes were incubated at 55 °C for 1 h in a water bath.

To determine the detection limit, the experimental data were visualized using a line graph, and the point of intersection of the fluorescence graph with the threshold was found. The threshold signal was calculated as the fluorescence from the negative control tube (background) plus three times the standard deviation of the background [25].

## 5. Conclusions

This research reports a diagnostic approach based on the implementation of viral RNA detection by DNA nanosensors with fluorescence labels for the detection of viral infections, including parainfluenza virus type 3 and respiratory syncytial virus A.

Two similar DNA nanosensing systems were developed and tested: binary MB-DNSs and 6-arm deoxyribozyme DNMs. These DNA nanosensors both produce fluorescence signals but utilize different reporters. Due to sensitivity limitations, the binary MB-DNSs were combined with RT-PCR as an alternative method of visualizing the amplification. The multicomponent 6DNMs exceed the earlier presented 4DNMs’ demonstrated picomolar sensitivity and provide an opportunity for use in amplification-free approaches. This achievement is promising for the integration of multicomponent machines into laboratory diagnostic platforms for poorly equipped facilities. The experiments carried out on clinical samples confirmed the functionality of these systems in real-world applications, although with varying degrees of success. This study’s small sample size reflects practical constraints in accessing clinical materials during the research period. While preliminary, these results validate the feasibility of 6DNMs for viral RNA detection. Larger-scale validation is planned pending collaboration with clinical partners to secure additional samples. The improvement of the DNM’s sensitivity remains essential for the successful integration of the system into clinical practice, which our team will pursue in future experiments. Both sensor types show potential for simplifying the detection of nucleic acids and can be recommended for further development in industry.

## Figures and Tables

**Table 1:** Comparison of molecular beacon-based DNA nanosensor, 6-arm DNA nanomachine’s characteristics, and RT-qPCR.

Criteria	MB-Based DNA Nanosensors	Deoxyribozyme DNA Nanomachines	RT-qPCR
**Detection speed**	15–30 min	30–180 min	90–120 min
**Ease of development**	Computer tools are used for designing Based on the principle of complementarity	Computer tools are used for designing Based on the principle of complementarity	Computer tools are used to select primers based on the principle of complementarity The amplification and visualization protocols are additionally selected, often empirically
**Sensitivity**	Approximately 1–3 nM	Approximately 5–10 pM	Approximately 3 attoM
**Specificity**	Specific	Specific	Specific
**Complexity**	Does not require assembly step, easy to design	The assembly is necessary, the reaction is simple, require addition equipment to incubate samples, easy to design	Method requires specific expensive equipment, reagents and enzymes, laboratory condition, highly qualified personnel
**Cost of the analysis**	USD 0.35 per reaction	USD 0.6 per reaction	USD 1.7 per reaction
**Competitiveness with existing methods**	Cannot achieve the same sensitivity as PCR; works in combination as an alternative to visualization and recognition of specific PCR product	Cannot achieve the same sensitivity as PCR but able to achieve amplification-free detection of fM concentrations of ssRNA	Remains the gold standard for accurate quantification of nucleic acids. The success of detection strongly depends on the stages of sample preparation and amplification
**Feasibility of viral RNA detection**	Effectively detects short ssDNA, PCR products	Detects short DNA analyte as well as long viral RNA samples	Detects DNA/RNA after amplification

## Data Availability

Data is contained within the article and Supplementary Materials.

## Supplementary Materials

The following supporting information can be downloaded at https://www.mdpi.com/article/10.3390/ijms26083652/s1.
