# Supplementary material for: Multicomponent DNA Nanomachines for Amplification-Free Viral RNA Detection
Source: Int J Mol Sci. 2025 Apr 12;26(8):3652. doi: 10.3390/ijms26083652 (PMC12026728; doi:10.3390/ijms26083652)
Supplement: Supplementary file 1 [file ijms-26-03652-s001.zip › ijms-3538794-supplementary.pdf]

International Journal of *Molecular Sciences*

MDPI

Supplementary Material

Multicomponent DNA Nanomachines for Amplification-free Viral RNA Detection

Valeria V. Solyanikova [1,†], Daria A. Gorbenko [1,†], Valeriya V. Zryacheva [2], Anna A. Shtro [2,*] and Maria S. Rubel [1,3]

[1] DNA-Nanosensoric Diagnostic Lab., ITMO University, 9 Lomonosova St., 191002 St. Petersburg, Russia; v_solyanikova@mail.ru (V.V.S.); daryarogova7@gmail.com (D.A.G.); msrubel@itmo.ru (M.S.R.)
[2] Smorodintsev Research Institute of Influenza, 15/17 Prof. Popova St., 197022 St. Petersburg, Russia; Valerya-zr4@yandex.ru
[3] Amyloid Biology Lab., St. Petersburg State University, 7-9 Universitetskaya enb., 199034 St. Petersburg, Russia
* Correspondence: anna.shtro@influenza.spb.ru
† These authors contributed equally to this work.

Table of content:

1. Oligonucleotides used in this study, **Table S1, S2, S3**
2. Analysis of amplification products, **Table S4**
3. Assembly of the 4DNM, **Figure S1**
4. Titration assay, **Figures S2 and S3, Table S5**
5. Limit of detection of 4DNMs, **Figure S4**
6. **Raw data for LODs, Table S6, S7, S8, S9 and S10**

1. Oligonucleotides used in this study.

Table S1. Sequence of MB-DNSs

Oligonucleotide name	Sequence 5'-3'
HPIV molecular beacon-based DNA nanosensor (HPIV MB-DNS)	
HPIV_an	ATCAAACAAGAAGGTAGACTCTTTGCAAAAATGACATACAAAAT GAGAGCTACACAGGTTTTATCAGAGACACTACTTGCAAACAATA TAGGAAAATTCTTTCAAGAAAATGGGATGGT
HPIV_m1	TATATTGTTTGC HEG **TATGTTAACAAC**
HPIV_f1	**GATCTATTG** HEG AAGTAGTGTCTCTGATC
RSV molecular beacon-based DNA nanosensor (RSV MB-DNS)	
RSV_an	TAAAGAGTGTTGTTAGTGGAGATATACTATCATATTCTATAGCTG GACGTAATGAAGTTTTCAGCAATAAACTTATAAATCATAAGCATA TGAACATCTTAAAGTGGTTC

RSV_m1	CTGAAAACTTC HEG **TATGTTAACTAC**
RSV_f1	**GATCTATTG** HEG ATTACGTCCAGCTATAGAAT
Molecular beacon	
MB	FAM*-CGCGTTAACATACAATAGATCGCG-BHQ

*FAM - 5-Carboxyfluorescein; BHQ - Black Hole Quencher; BHQ1 - Black Hole Quencher 1; HEG - Hexaethylene glycol linker. Green and red colors correspond to the m- and f-binding arms of the MB-DNSs. The complementary to the molecular beacon parts of the sensors are highlighted in bold.

Table S2. Sequence of DNMs

Oligonucleotide name	Sequence 5'-3'
HPIV 6-arm DNA nanomachine (HPIV 6DNM) and 4-arm DNA nanomachine (HPIV 4DNM)	
HPIV_an6	GAAAAGGAGATCAAACAAGAAGGTAGACTC<br>TTTGCAAAAATGACATACAAAATGAGAGCT<br>ACACAGGTTTTATCAGAGACACTACTTGCA<br>AACAATATAGGAAAATTCTTTCAAGAAAAT<br>GGGATGGTGAAGGGAGAGATTGAATTACTT<br>AAGAGATTAACAACTATATCAATATCAGGA
HPIV_Tile	CTCTACTGACGTGCCGtttCTTATGTATACGACGCTAACT<br>CAACCACGGTGAAATTTGACTTTACTTTTCTCCTCTAGAAGTTGA<br>AAATGGGCGAATAATTCT<br>CCTAAAACTAACTTGAACTACtttCTCTACTGACGTGCCG
HPIV_a1t1	TCCTGATATTGATATAGTTGTTAATCTCTTtttttt*GTAGTTCAAGTTAGTTTTAGG*
HPIV_a2t2a5	AAGTAATTCAATCTCTCCCTTCACCATCCCtttttt*AGAATTATTCGCCCATTTTCAACTTCTAGAGGAGAAAAGTAAAGTCAAATTTCACCGTGGTTG*ttttttAGCTCTCATTTTGTATGTCATTTTTGCAAA
HPIV_a3c	ATTTTCTTGAAAGAATTTTCCTATATTGTT**ACAACGAGAGGAAACCTT**
HPIV_ca4t3a6	**TGCCCAGGGAGGCTAGCT**TGCAAGTAGTGTCTCTGATAAAACCTGTGTtttttt*AGTTAGCGTCGTATACATAAG*ttttttGAGTCTACCTTCTTGTTTGATCTCCTTTTC
HPIV_4arm_ca4t	**TGCCCAGGGAGGCTAGCT**TGCAAGTAGTGTCTCTGATAAAACCTGTGT*ttttttCAACCACGGTGAAATTTGACTTTACTTTTCTCCTCTAGAAGTTGAAAATGGGCGAATAATTCT*
RSV 6-arm DNA nanomachine (RSV 6DNM) and 4-arm DNA nanomachine (RSV 4DNM)	

RSV_an6	TTTCT TTGTTACCCTATAACAAAAAAAGGAATTAA TACTGCATTGTCTAAATTAAAGAGTGTTGT TAGTGGAGATATACTATCATATTCTATAGC TGGACGTAATGAAGTTTTCAGCAATAAACT TATAAATCATAAGCATATGAACATCTTAAA GTGGTTCAATCATGTTTTAAATTTCAGATC AACAG
RSV_Tile	CTCTACTGACGTGCCGtttCTTATGTATACGACGCTAACT CAAGCACGGTGAAATTTGACTTTACTTTAGTAGACTAGTTGATGA AAATGGCGCTATAATTCT CCTTGATCTATGTCGATATACtttCTCTACTGACGTGCCG
RSV_a1t1	GATCTGAAATTTAAAACATGATTGAACCACtttttt*GTATATCGACATA GATCAAGG*
RSV_a2t2a5	TTTAAGATGTTCATATGCTTATGATTTATAttttt*AGAATTATAGCGCC ATTTTCATCAACTAGTCTACTAAAGTAAAGTCAAATTTCACCGTGCTTGt* tttttACAACACTCTTTAATTTAGACAATGCAGTA
RSV_a3c	AGTTTATTGCTGAAAACTTCATTACGTCCA**ACAACGAGAGGAAA CCTT**
RSV_ca4t3a6	**TGCCCAGGGAGGCTAGCT**GCTATAGAATATGATAGTATATCTC CACTAtttttt*AGTTAGCGTCGTATACATAAG*ttttttTTAATTCCTTTTTTTG TTATAGGGTAACAA
RSV_ 4arm_ca4t	**TGCCCAGGGAGGCTAGCT**GCTATAGAATATGATAGTATATCTC CACTA*ttttttAAGCACGGTGAAATTTGACTTTACTTTAGTAGACTAGTTGA TGAAAATGGCGCTATAATTC*
Hook	AACTTCTACAATGTACCGtttttCGGCACGTCAGTAGAG
Fluorescent substrate	
F-sub	CGGTACATTGTAGAAGTTAAGGTTFAMTCCTCguCCCTGGGCABH Q1

*FAM – 5-Carboxyfluorescein; BHQ – Black Hole Quencher; BHQ1 – Black Hole Quencher 1. Green, yellow amd blue colors correspond to analyte-binding and tile-forming arms of the 6DNMs. The core-forming fragments are highlighted in bold. Tile and linker fragments are in italics.

Table S3. Sequence of primers

Oligonucleotide name	Sequence 5'-3'
PCR primers for HPIV	
HPIV_F	AGGAGATCAAACAAGAAGGTAGAC
HPIV_R	CTTCATTATATCGTGGAACTCCTGA
PCR primers for RSV	
RSV_F	TTGATACCCTTTCTTTGTTACCCT

RSV_R	GATGAGGATAAGTAGATTCCACCA

2. Analysis of amplification products

2.1. PCR-products detection.

Extracted from the viral particles genome RNA was used as a template for the PCR. Amplification of the target was done according to the protocol (Table S4). The visualization performed in 2% agarose gel electrophoresis in TBE buffer. For the reverse transcription, 500 ng of RNA from each virus were taken. For the PCR step, RT products diluted two times were taken. The detection obtained after RT-PCR amplicons was done according to the same protocol that was used for detection with synthetic analyte. But before detection the purification of amplicons and denaturation at 95 °C for 5 min was done.

Table S4. PCR modes for HPIV, RSV fragments amplification

Stage	T, °C	Time	Cycles
Initial denaturation	95	5′	1
Denaturation	95	30″	35
Annealing	57	20″	
Elongation	72	30″	
Elongation	72	5′	1

3. Assembly of the 4DNM in gel.

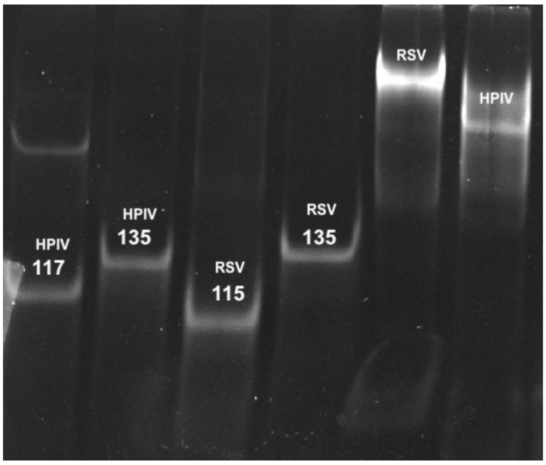

Figure S1. 8% PAGE electrophoresis of 4DNMs assembly: 1 – HPIV_4arm_ca4t (117 bp); 2 – HPIV_a2t2a5 strand (135 bp); 3 – RSV_4arm_ca4t (115 bp); 2 – RSV_a2t2a5 strand (135 bp); 5 – assembled RSV 4DNM; 6 - assembled HPIV 4DNM

The HPIV_4arm_ca4t formed the dimeric structure that caused the uncomplete folding of the DNM and may be a reason of lower LOD.

4. Titration assay.

For the implementation of the detection experiments, we determined the free arm-to-machine concentration ratio when the detection signal is the highest but the background is low at the same time. All of the 6DNMs detection experiments were conducted based on these ratios.

Different concentration ratios of the assembled DNA machine and free arm were tested. The fluorescence signal was detected after 1 h incubation at 55 °C by a fluorimetry Tecan Spark multimode reader (Figure S1). Also the background to the F-

sub signal ratio was calculated (Figure S2). The obtained fluorescence data was normalized as well as background to the F-sub signal ratio and summarized to select the best machine to arm ratio. This procedure was done for all developed multicomponent DNA nanomachines. The data after normalization and calculation are presented in Table S5.

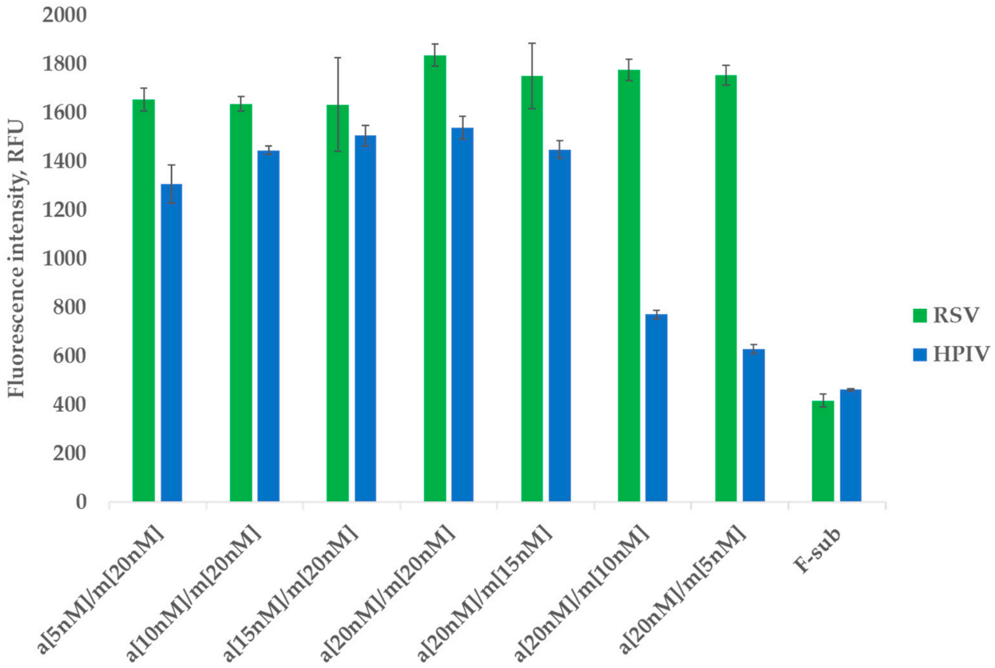

Figure S2. The fluorescence signal detected with HPIV 6DNM (blue) and RSV 6DNM (green) after 1 h incubation at 55 °C. a – arm 3; m – assembled machine parts with tile

As a result of the experiment, several concentration ratios for both HPIV 6DNM and RSV 6DNM show a high signal, but the ratio of the fluorescence signal of samples without analyte to the signal of F-sub must also be taken into account.

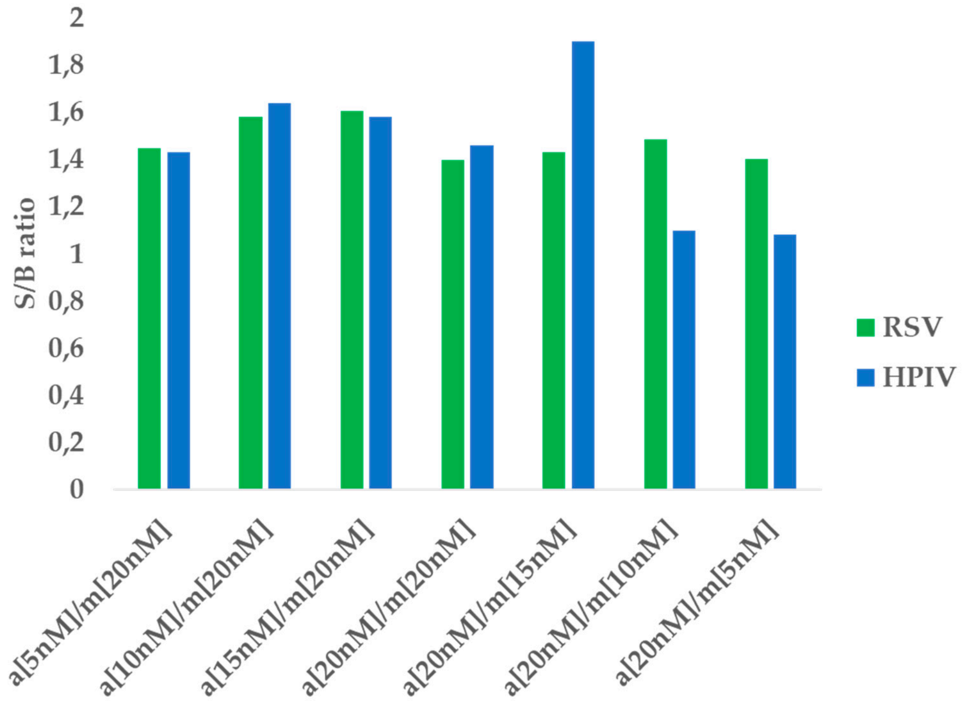

Figure S3. Results of the background to the f-sub signal ratio. HPIV 6DNM results are blue bars, RSV 6DNM results - green. a – arm 3; m – assembled machine with tile

Lowest signal-to-background (S/B) ratio in the HPIV 6DNM experiment at 20 nM arm per 10 nM machine. In the experiment with the RSV 6DNM, the best performance was in the case of the ratio of concentrations of 20 nM free arm to 20 nM machine and 20 nM free arm to 5 nM machine. To determine the best ratio, the stage of linear normalization of the experimental results was carried out. After summing the fluorescence intensity values and signal-to-background ratios, the optimal variant was selected for each DNA nanomachine (Table S5).

Table S5. Titration assay data normalization and calculation

Arms ratio	Fluorescence (mean), RFU	Normalized fluorescence	S/B ratio	Normalized S/B ratio	Sum
RSV					
a[5nM]/ m[20nM]	1654	0,87	1,45	0,74	1,13
a[10nM]/ m[20nM]	1636	0,86	1,58	0,96	0,90
a[15nM]/ m[20nM]	1633	0,86	1,61	1	0,86

a[20nM]/ m[20nM]	1836	1	1,40	0,65	**1,35**
a[20nM]/ m[15nM]	1750	0,94	1,43	0,71	1,23
a[20nM]/ m[10nM]	1776	0,96	1,48	0,80	1,16
a[20nM]/ m[5nM]	1754	0,94	1,40	0,66	1,28
HPIV					
a[5nM]/ m[20nM]	1307,33	0,75	1,43	0,43	1,32
a[10nM]/ m[20nM]	1445,33	0,90	1,64	0,68	1,21
a[15nM]/ m[20nM]	1505,33	0,96	1,58	0,61	1,35
a[20nM]/ m[20nM]	1538,33	1	1,46	0,46	**1,54**
a[20nM]/ m[15nM]	1448	0,90	1,90	1	0,90
a[20nM]/ m[10nM]	770,67	0,16	1,1	0,02	1
a[20nM]/ m[5nM]	627,67	0	1,08	0	1,13

As a result, for HPIV and RSV 6-arm DNA nanomachines the best arms ratio is 20 nM free arm to 20 nM of machine. These arms ratios were used for detection experiments with synthetic analytes and viral RNA.

5. Limit-of-detection of 4DNMs

A)

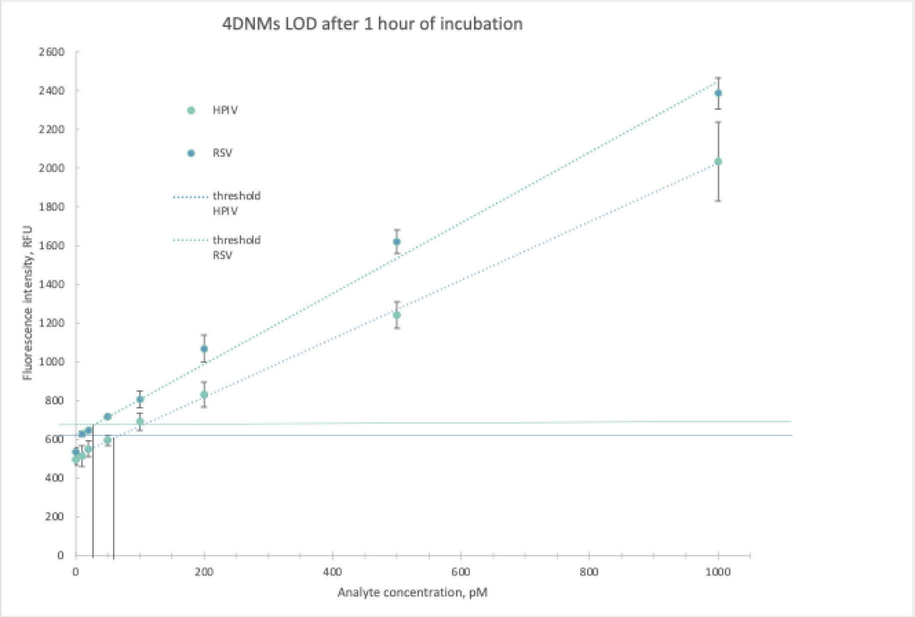

B)

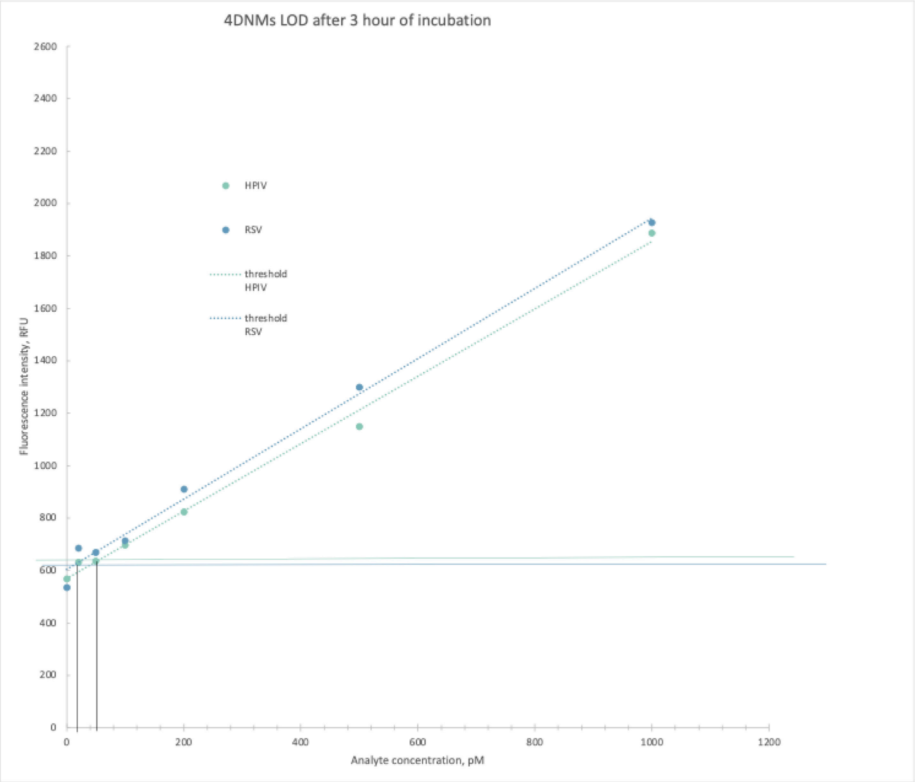

Figure S4. Limit of detection estimated for the synthetic analyte by 4DNMs. A) HPIV and RSV 6DNMs after 1 h of incubation; B) HPIV and RSV 4DNMs after 3 h of incubation. The blue color corresponds to the HPIV 4DNM result; The green color corresponds to the RSV 4DNM result. Dotted lines are the linear trendlines. The black

lines indicate the detection limits for each DNM. Data represent mean ± SD of triplicate experimental measurements.

6. Raw data for LODs

A)

	Analyte concentration, nM									
	**MB**	**0**	**1**	**5**	**10**	**15**	**20**	**30**	**50**	**100**
#1	53	60	71	76	112	87	102	89	105	94
#2	43	52	78	79	103	79	101	78	101	102
#3	55	67	72	67	102	86	98	86	88	85
Mean	50,33	59,67	73,67	74	105,67	84	100,33	84,33	98	93,67
SD	6,43	7,51	3,79	6,24	5,51	4,36	2,08	5,69	8,89	8,50

B)

	Analyte concentration, nM									
	**MB**	**0**	**1**	**5**	**10**	**15**	**20**	**30**	**50**	**100**
#1	42	51	58	63	74	75	93	84	102	93
#2	45	52	63	65	69	53	95	69	74	72
#3	41	56	56	64	74	53	91	59	71	67
Mean	42,67	53	59	64	72,33	60,33	93	70,67	82,33	77,33
SD	2,08	2,65	3,61	1	2,89	12,70	2,83	12,58	17,10	13,80

Table S6. Limit of detection determination data estimated for the synthetic analyte by MB-based DNSs. A) HPIV-DNS; B) RSV-DNS.

A)

	Analyte concentration, pM											
	**blank**	**0**	**1**	**2**	**5**	**10**	**20**	**50**	**100**	**200**	**500**	**1000**
#1	219	269	306	267	249	360	258	286	406	472	1038	1008
#2	285	265	327	293	236	358	271	303	404	524	1032	1000
#3	211	252	246	224	238	294	198	282	405	474	877	862
#4	233	277	364	358	341	400	351	438	565	769	1161	1211
#5	215	263	344	351	326	409	421	424	575	793	1197	1096
#6	228	260	299	332	317	382	347	407	547	676	1169	1123
Mean	231,83	264,33	314,33	304,17	284,5	367,17	307,67	356,67	483,67	618	1079	1050
SD	27,30	8,43	41,19	52,53	48,47	41,32	80,13	73,66	86,64	146,75	121,20	120,81

B)

	Analyte concentration, pM											
	**blank**	**0**	**1**	**2**	**5**	**10**	**20**	**50**	**100**	**200**	**500**	**1000**
#1	361	389	398	386	425	469	441	582	859	1380	2054	2005
#2	359	379	390	375	428	453	452	598	825	1244	1916	2048
#3	343	367	355	324	364	395	356	577	735	1219	1812	2022
#4	314	387	509	527	534	434	542	632	1049	1577	1995	2307
#5	308	377	500	456	393	479	522	595	1057	1531	1952	2345
#6	309	397	485	461	527	540	533	628	1012	1445	1761	2211
Mean	332,33	382,67	439,5	421,5	445,17	461,67	474,33	602	922,83	1399,33	1915	2156,33
SD	24,98	10,54	66,14	73,23	70,14	48,53	71,98	23,09	134,76	146,98	110,83	150,98

Table S7. Limit of detection determination data estimated for the synthetic analyte by HPIV 6DNM. A) LOD after 1 hour of incubation; B) LOD after 3 hours of incubation.

A)

	Analyte concentration, pM										
	**blank**	**0**	**2**	**5**	**10**	**20**	**50**	**100**	**200**	**500**	**1000**
#1	242	389	458	398	586	467	477	898	1223	1745	2643
#2	249	405	471	393	466	472	518	886	1231	1742	2640
#3	225	378	306	368	361	422	480	832	1083	1682	2424
#4	229	422	482	462	536	547	522	997	1298	1932	2267
#5	231	406	464	497	532	607	525	812	1222	1772	1896
#6	212	381	336	371	473	389	413	724	1118	1569	1777
Mean	231,33	396,83	419,5	414,83	492,33	484	489,17	858,17	1195,83	1740,33	2274,5
SD	13,01	17,03	77,30	52,64	78,19	80,50	42,96	92,14	79,86	118,76	369,48

B)

	Analyte concentration, pM										
	**blank**	**0**	**2**	**5**	**10**	**20**	**50**	**100**	**200**	**500**	**1000**
#1	322	556	596	642	626	749	771	1735	1628	1727	3032
#2	302	545	583	626	604	711	799	1566	1713	2068	3109
#3	358	516	364	559	578	516	701	1783	1766	1747	2742
#4	276	534	764	692	663	701	686	1594	2150	2307	2610
#5	258	567	676	693	585	672	632	1223	1561	1980	2100
#6	312	504	555	563	579	401	538	1122	1316	1665	2072
Mean	304,6667	537	589,6667	629,1667	605,8333	625	687,8333	1503,833	1689	1915,667	2610,833
SD	35,22878	23,93324	134,2068	59,15883	33,48681	136,2454	94,88818	271,3046	275,0491	247,9368	445,8809

Table S8. Limit of detection determination data estimated for the synthetic analyte by RSV 6DNM. A) LOD after 1 hour of incubation; B) LOD after 3 hours of incubation.

A)

	Analyte concentration, pM						
	1000	500	200	100	50	20	0
#1	2655.67	2692.67	1991.67	1312.33	892.67	712.67	533.67
#2	2731.33	2763.67	1970.00	1313.00	948.67	720.00	556.33
#3	2963.00	2843.33	1886.00	1275.33	885.00	702.33	517.67
Mean	2783.33	2766.56	1949.22	1300.22	908.78	711.67	535.89
SD	160.13	75.37	55.81	21.56	34.76	8.88	19.43

B)

	Analyte concentration, pM							
	1000	500	200	100	50	20	10	blank
#1	2475.67	1688.67	1116.67	855.67	729.67	652.33	641.67	525.00
#2	2325.33	1598.00	1097.67	770.67	711.33	632.67	632.33	518.00
#3	2355.33	1572.33	989.00	796.00	717.33	650.33	612.33	559.67
Mean	2385.00	1620.00	1068.00	807.00	719.00	645.00	629.00	534.00
SD	79.56	61.12	68.88	43.64	9.35	10.82	14.99	22.31

Table S9. Limit of detection determination data estimated for the synthetic analyte by RSV 4DNM. A) LOD after 1 hour of incubation; B) LOD after 3 hours of incubation.

A)

	Analyte concentration, pM						
	1000	500	200	100	50	20	0
#1	1903.00	1184.67	851.00	671.00	628.67	622.00	554.33
#2	1746.33	1023.67	788.00	688.00	613.00	596.00	545.33
#3	2030.33	1237.33	829.00	731.67	664.00	676.67	605.33
Mean	1893.22	1148.56	822.67	696.89	635.22	631.56	568.33
SD	142.25	111.32	31.97	31.29	26.12	41.17	32.36

B)

	Analyte concentration, pM							
	1000	500	200	100	50	20	10	blank
#1	1851.00	1246.00	847.00	708.33	566.33	544.33	537.00	488.67
#2	1996.00	1195.00	786.00	639.50	603.00	513.00	452.33	473.33
#3	2252.00	1291.67	876.67	725.33	615.33	594.33	554.00	531.00
Mean	2033.00	1243.00	831.00	691.00	595.00	551.00	514.00	498.00
SD	203.04	48.36	46.23	45.45	25.49	41.02	54.46	29.87

Table S10. Limit of detection determination data estimated for the synthetic analyte by HPIV 4DNM. A) LOD after 1 hour of incubation; B) LOD after 3 hours of incubation.
